# Intratumoral anti-HuD immunotoxin therapy for small cell lung cancer and neuroblastoma

**DOI:** 10.1186/s13045-014-0091-3

**Published:** 2014-12-19

**Authors:** Debra Ehrlich, Bo Wang, Wei Lu, Peter Dowling, Ruirong Yuan

**Affiliations:** Department of Neurology and Neurosciences, Rutgers NJMS, Newark, NJ USA; Neurology Service, VA Medical Center, East Orange, NJ USA

## Abstract

**Background:**

Most patients with small cell lung cancer (SCLC) or neuroblastoma (NB) already show clinically detectable metastases at diagnosis and have an extremely poor prognosis even when treated with combined modalities. The HuD-antigen is a neuronal RNA-binding protein that is expressed in 100% of SCLC tumor cells and over 50% of neuroblastoma cells. The correlation between high titers of circulating anti-HuD antibodies in patients and spontaneous tumor remission suggests that the HuD-antigen might be a potential molecular target for immunotherapy.

**Methods:**

We have constructed a new antibody-toxin compound (called BW-2) by assembling a mouse anti-human-HuD monoclonal antibody onto streptavidin/saporin complexes.

**Results:**

We found that the immunotoxin BW-2 specifically killed HuD-positive human SCLC and NB cancer cells at very low concentrations in vitro. Moreover, intratumoral immunotoxin therapy in a nude mouse model of human SCLC (n = 6) significantly reduced local tumor progression without causing toxicity. When the same intratumoral immunotoxin protocol was applied to an immunocompetent A/J mouse model of NB, significant inhibition of local tumor growth was also observed. In neuroblastoma allografted A/J mice (n = 5) treated twice with intratumoral immunotoxin, significant tumor regression occurred in over 80% of the animals and their duration of tumor response was significantly prolonged.

**Conclusions:**

Our study suggests that anti-HuD based immunotoxin therapy may prove to be an effective alternative treatment for patients with SCLC and NB.

## Background

Both small cell lung cancer (SCLC) and neuroblastoma (NB) express high-levels of HuD protein. HuD is a 40 kD neuronal RNA-binding protein that is expressed in 100% of SCLC tumor cells and at least 50% of NB cells [1]. Most patients with SCLC or NB have disseminated disease at the time of diagnosis and the prognosis is usually poor despite aggressive multimodality treatment. New and effective therapies are needed to improve disease outcome in these patients. High polyclonal anti-HuD antibody titers are associated with occasional spontaneous remission in some SCLC patients, suggesting that the HuD-antigen might be a good molecular target for specific immunotherapy against HuD positive tumors [2].

Immunotoxins are unique proteins made by conjugating toxins to antigen specific antibodies that are designed to kill a targeted cell population [3]. Saporin, a ribosomal toxin, is a plant enzyme that irreversibly blocks protein synthesis. Antibodies conjugated to saporin have been employed for leukemia treatment and for pain control in neurologic disorders [4,5]. The majority of immunotoxins developed for cancer treatment have targeted leukemia and are administered intravenously [5,6]. Multiple doses are often required to achieve a therapeutic effect. Clinical success of systemic immunotoxin therapy in solid tumors has been largely unimpressive because of poor immunotoxin penetration into the tumor and due to toxin side effects, such as vascular leak syndrome [7]. Little is known regarding the efficacy of intratumoral (i.t.) immunotoxin therapy on solid tumors.

In this report we describe a new antibody-toxin compound (BW-2), which is constructed by attaching a biotinylated anti-HuD monoclonal antibody (mAb) onto a streptavidin-saporin complex. We found that this immunotoxin aggressively killed HuD-positive SCLC and NB cells in vitro with high specificity. Intratumoral injection of the immunotoxin greatly inhibited tumor progression without inducing toxicity in a nude mouse model of human SCLC. Furthermore, intratumoral immunotoxin induced significant tumor regression in 80% of immunocompetent neuroblastoma allografted A/J mice and significantly prolonged duration of tumor response. This new compound may offer a therapeutic option with significant potential for patients with tumors that express the HuD protein.

## Results

### SCLC and neuroblastoma cell line HuD-protein detection by anti-HuD mAb

**Figure 1:**
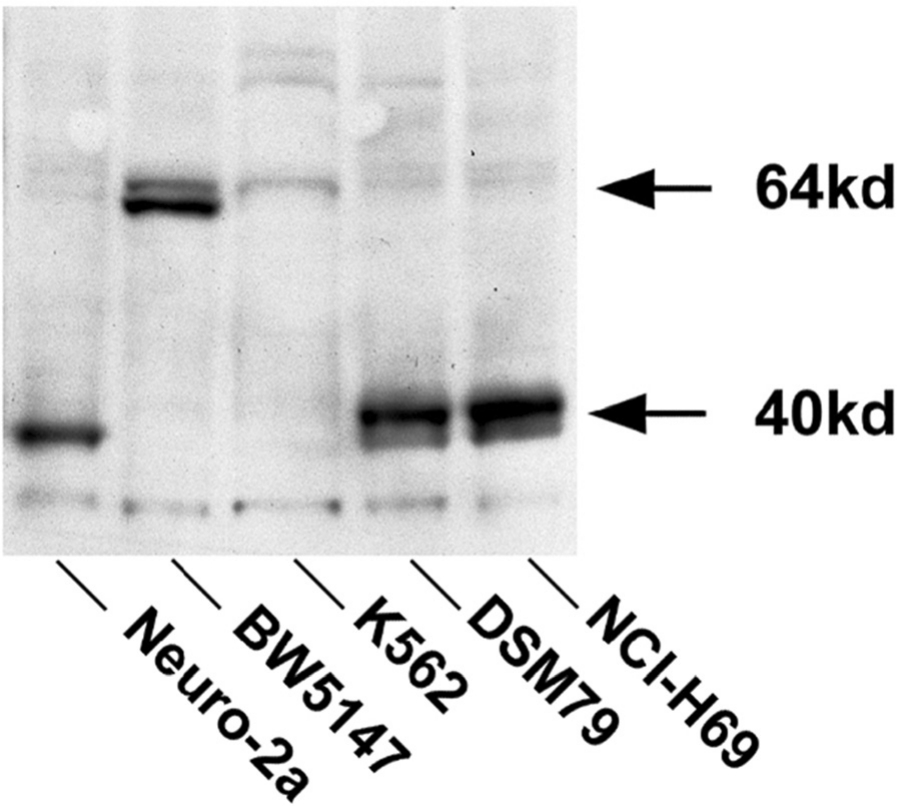
**Western blot analysis of HuD proteins in SCLC, neuroblastoma, and leukemia/lymphoma cell lines.** Protein extracts from cell lysates were subjected to SDS-polyacrylamide gel electrophoresis (SDS-PAGE) on a NuPAGE™ 4-12% Bis-Tris gel, transferred to nitrocellulose membrane. The membrane was probed with anti-HuD mAb (16A11) and a HRP-conjugated goat anti-mouse IgG detection system. The neuroblastoma (Neuro-2a) and SCLC (NCI-H69, DSM79) extracts demonstrated strong bands at 40 kD, whereas two control cell line (BW5147, K562) extracts were devoid of signal.

To assure that mouse mAb 16A11 was specific for HuD-antigen in both human and mouse HuD-positive cancer cells, we first tested the reactivity of the antibody to cell extracts obtained from SCLC, neuroblastoma, and extracts those extracted from leukemia and mouse T lymphoma control cell lines by Western blot. Figure 1 shows that human SCLC (NCI-H69, DMS79) and mouse neuroblastoma neuro-2a cell extracts strongly express HuD (~39-40 kD) whereas control cell lines (BW5147 and K562) failed to express HuD antigen at the expected molecular weight. All cell lines displayed faint nonspecific binding at ~64 kD, but lymphoma control cell line BW5147 showed stronger signal compared to all other cell lines.

### BW-2 immunotoxin specifically kills Hu-D positive tumor cells

**Figure 2:**
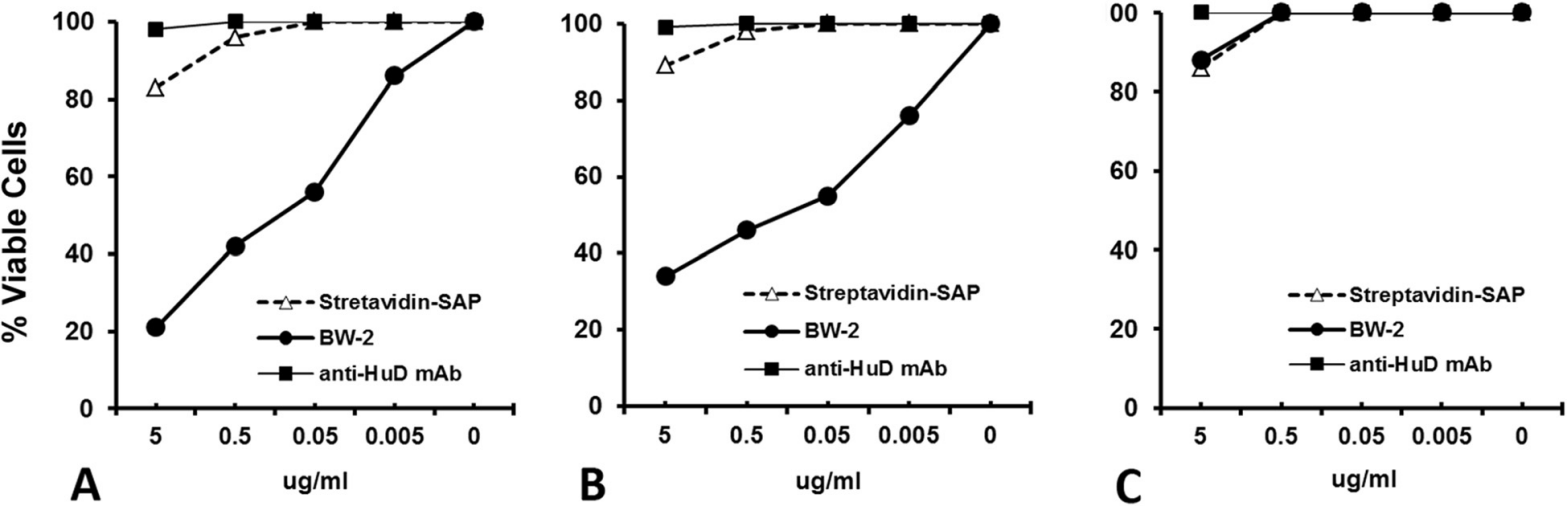
**Immunotoxin BW-2 efficiently kills SCLC and neuroblastoma cancer cells.** BW-2 kills **(A)** HuD-positive H69 cells and **(B)** neuro-2a, whereas no cytotoxicity occurred in the control **(C)** HuD-negative K562 cells. Log dilutions of BW-2 (0–5 μg/ml) were added to the cell culture wells (5 × 10^4^ cells/well) and incubated at 37°C for 72 hr. Exposure to equal molar concentrations of streptavidin-saporin (Streptavidin-SP) alone or mAb (16A11) alone served as controls. BW-2 efficiently killed the targeted H69 and neuro-2a cancer cells with great potency *(*P < 0.005*).* SAP-streptavidin alone or 16A11 mAb alone induced only mild non-specific killing at higher concentrations. Data represents the mean of three tests in a single representative experiment repeated 3 times.

The killing effect of BW-2 was quantified in HuD-positive NCI-H69, Neuro-2a, and HuD-negative K562 tumor cell lines. Figure 2 shows that the BW-2 construct killed the targeted NCI-H69 SCLC (Figure 2A) and neuro-2a (Figure 2B) cells with great potency (ED_50_ < 0.5 μg/ml). In contrast, the saporin-streptavidin complex alone or mAb alone exhibited limited non-specific killing only at much higher concentrations (P < 0.05). The addition of non-biotinylated mAb at a 20-fold excess only partially blocked cell killing by BW-2. Adding either streptavidin or biotin alone to NCI-H69 or neuro-2a cell cultures failed to block cell killing induced by BW-2. Minimal non-specific cytotoxicity was induced in HuD-negative control cells, but again only at much higher concentrations of BW-2 (2–5 μg/ml) (Figure 2A). Trypan blue staining for cell death detection as well as the Cell Titer 96 Proliferation Assay were utilized to confirm cell viability and all tests showed similar results (data not shown).

### Anti-HuD immunotoxin inhibits tumor growth and prolongs duration of tumor response in tumor xenografted mice

**Figure 3:**
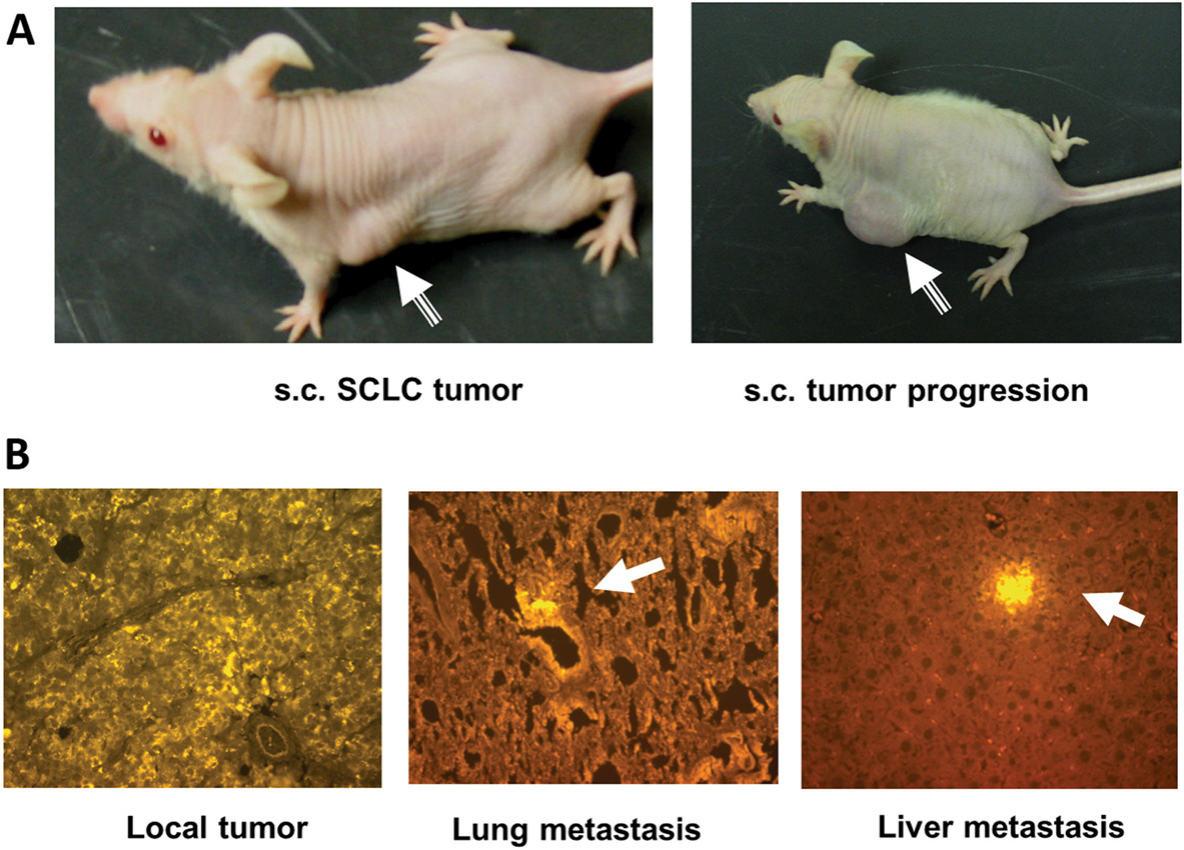
**Nude mouse model of human SCLC and pathology.** Viable H69 SCLC cells (1x10^7^/100 μl PBS) were subcutaneously implanted into the flank of nude mice. **(A)** Subcutaneous tumor nodules developed and progressed rapidly after s.c. implantation and multiple organ cancer metastases occurred when the s.c. tumor reached a certain size (~1000 mm^3^). **(B)** The metastasized cancer cells were easily detected by immunoreaction with biotinylated anti-HuD mAb and Cy3 fluorescent detection.

Nude mouse models of human SCLC have been used extensively by many investigators [8,9] and tumor xenografts in the nude mouse usually develop within 2 weeks after subcutaneous inoculation of NCI-H69 cancer cells. In our experiments, the tumor mass expanded to 300-500 mm^3^ over 2–3 weeks after s.c. implantation of 1 × 10^7^ viable tumor cells into the flanks of nude mice. Tumor-grafted mice were capable of surviving for 6–8 weeks before they were overwhelmed by the tumor burden (Figure 3A). In contrast to common belief, we found that after s.c. tumor implantation, significant tumor metastasis developed in multiple organs (lung, liver, and bone marrow) after the local tumor reached 1000–1500 mm^3^ and the metastasized cancer cells were easily detected by immunoreaction with biotinylated anti-HuD mAb and Cy3 fluorescent detection (Figure 3B).

**Figure 4:**
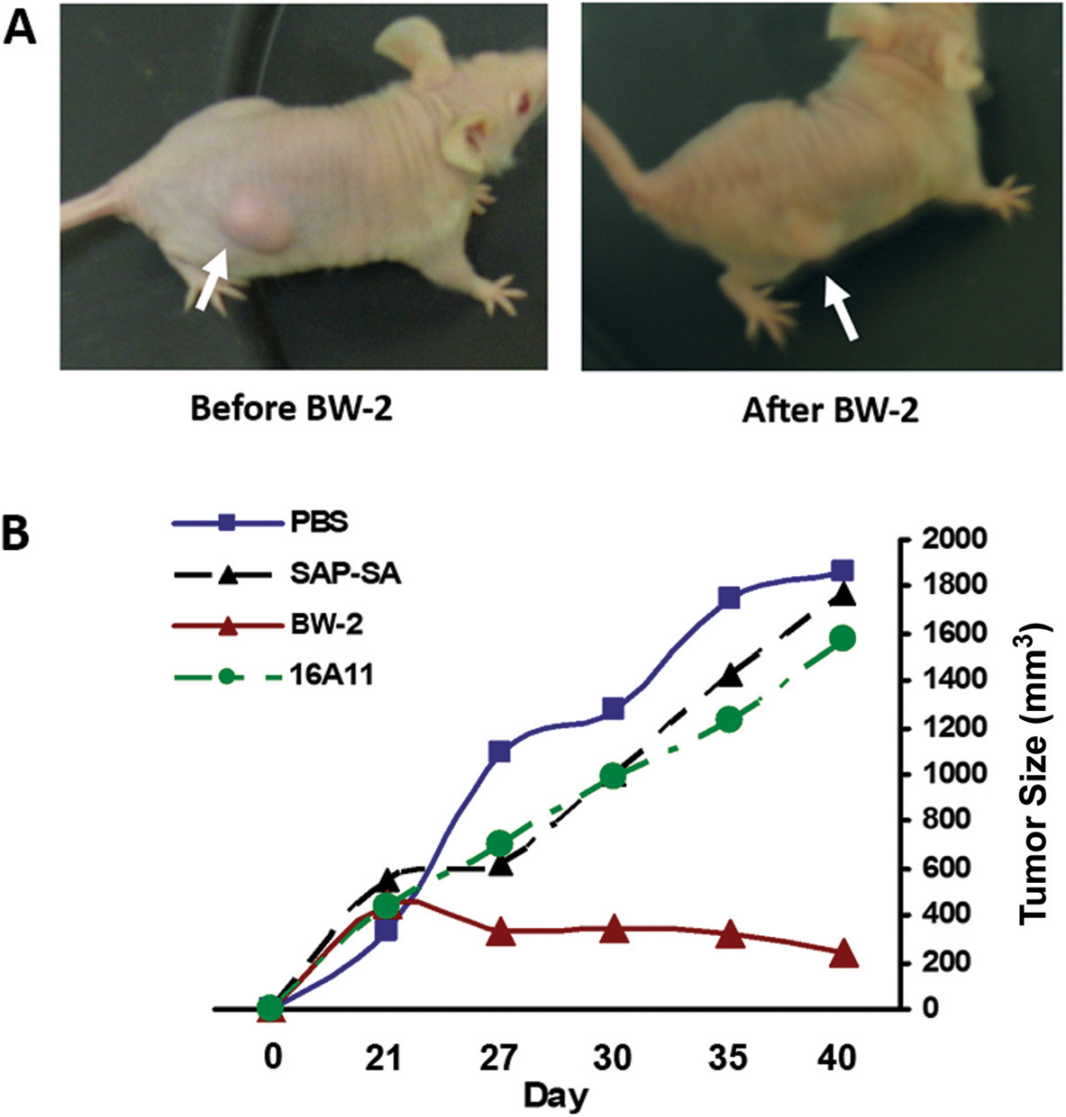
**Intratumorally injected immunotoxin BW-2 inhibits growth in HuD-positive H69 SCLC tumor xenografted mice.** SCLC tumor xenografted nude mice (n = 6) were treated with one time intratumoral immunotoxin, or an equivalent dose of mAb alone, saporin toxin alone, or saline. **A)** Tumor size in BW-2 treated mice remained static or actually regressed for ~3 weeks. **B)** The control mice exhibited rapidly progressive increases in tumor volume and were overwhelmed by large tumor burden as well as metastases.

To determine whether intratumoral BW-2 affected tumor growth, the tumor xenografted nude mice were treated once with immunotoxin (BW-2 at 1 mg/kg) injected directly into the s.c. tumor. Control tumor xenografted mice received equivalent subcutaneous injections of pure anti-HuD mAb alone, saporin toxin alone, or sham treatment with saline. The tumor shrinking effect of immunotoxin treatment was clearly visible as control mice continued to demonstrate rapid tumor volume expansion whereas the primary tumors in the BW-2 treated animals remained static or actually regressed for ~3 weeks (Figure 4A/B). The residual tumor eventually re-grew into a larger mass 6–9 weeks after one time local immunotoxin treatment.

**Figure 5:**
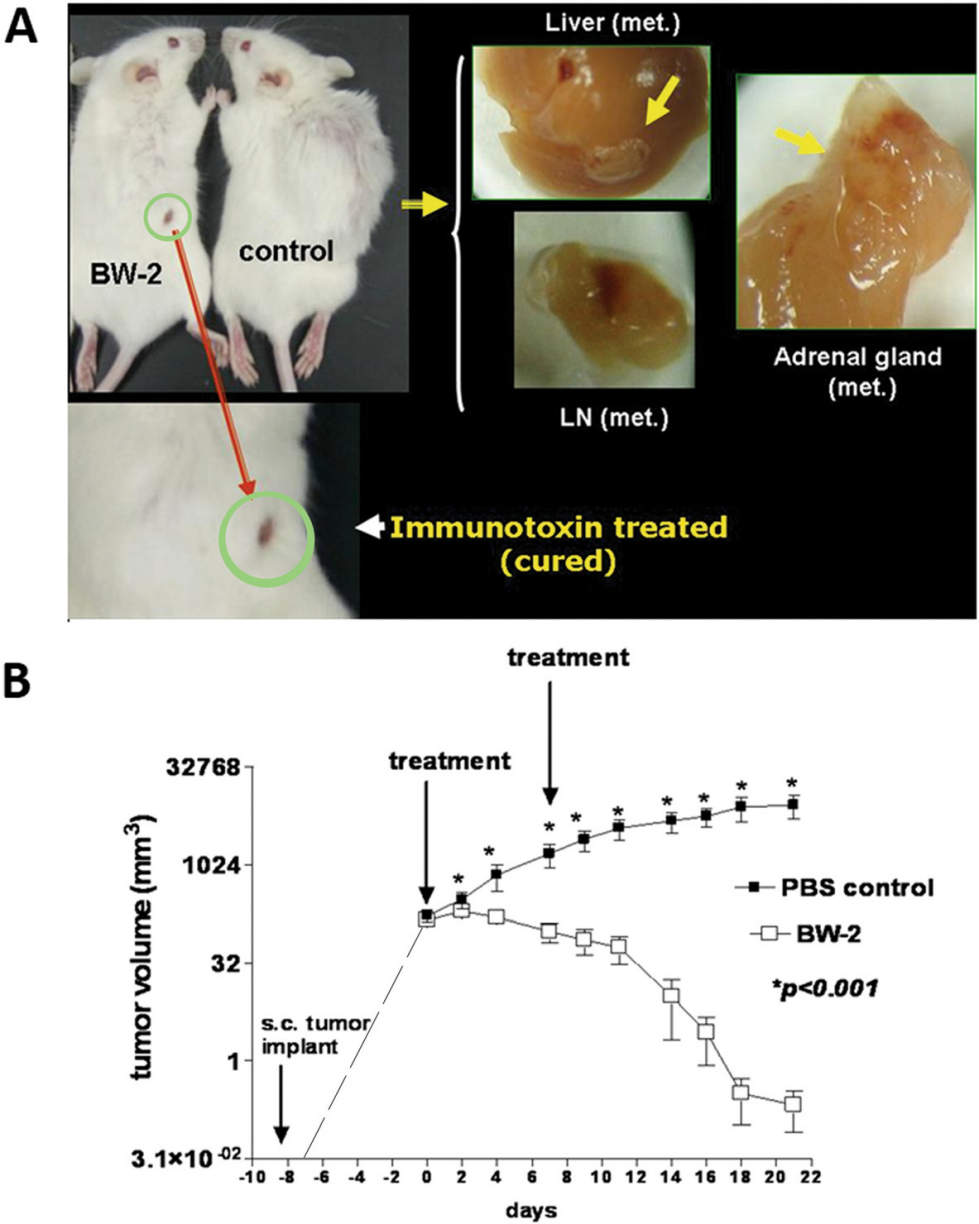
**Intratumorally injected BW-2 inhibits tumor growth and prolongs survival in HuD-positive neuroblastoma allografted mice. A)** Two weekly doses of intratumoral BW-2 treatment in neuro-2a allografted A/J mice (n = 5) significantly inhibited local tumor growth as compared to PBS treated controls. In BW-2 treated mice, a temporary loss of fur was observed at the injection site while significant local tumor growth was observed in control mice. Major organs in perfused animals were checked for evidence of metastasis. Metastatic tumors were found on the liver, lymph node and adrenal glands of some of the PBS treated animals. **B)** Significant tumor regression was observed in more than 80% of studied animals receiving BW-2 (P < 0.001).

We hypothesized that intratumoral immunotoxin therapy for SCLC or NB might be more effective in immunocompetent mice because the immunotoxin-induced tumor cell death would release abundant tumor antigens, which could potentially activate infiltrating immune cells locally to kill residual tumor cells and prevent distant metastasis. Because a mouse model of SCLC in immunocompetent mice is not available, the mouse model of HuD-positive neuro-2a NB in immunocompetent A/J mice was used for this study. The neuro-2a cell is a subclone of a murine neuroblastoma that was harvested from a spontaneously developed tumor in A/J mice. The neuro-2a implanted A/J mouse model has been used previously for studying efficacy of DNA vaccines [10]. As shown in Figure 5, more marked efficacy of BW-2 was observed in neuro-2a tumor allografted immunocompetent A/J mice. In BW-2 treated mice, a temporary loss of fur was observed at the injection site. Fur began to grow back within a few weeks after the final treatment. Otherwise, no other adverse effects were detected. Long-term follow-up over 7 weeks showed no local tumor relapse or distant metastasis in 80% of the BW-2 treated immunocompetent A/J mice. In contrast, the sham treated control mice had overwhelming local tumor growth. Additionally, multiple organs (brain, lung, liver, adrenal gland and bone marrow) had clearly detectable tumor metastases when examined either by direct tissue examination (Figure 5) or by immunohistochemistry.

## Discussion

SCLC and NB are both neoplasms of neuroendocrine origin. Reports of a strong correlation between the presence of anti-HuD antibodies and spontaneous remission of SCLC in some patients suggest that the HuD-antigen might be a specific molecular target for immunotherapy [11,12]. In this study, we report that a new anti-HuD immunotoxin, BW-2, effectively kills HuD-positive SCLC and NB cells in vitro. When the immunotoxin was injected directly into tumor sites, local tumor progression was profoundly inhibited with no evidence of significant toxicity detected in either tumor implanted mouse model. In fact, more than 80% of BW-2 treated neuro-2a allografted mice displayed significant tumor regression, and as a result, the tumor response in BW-2 treated tumor xenografted mice was substantially prolonged compared to all other control groups.

Until now, the majority of immunotoxins developed for cancer treatment have been used to target leukemia and have been administered intravenously [5,6]. Based on our in vitro studies, in which the anti-HuD mAb or BW-2 immunotoxin were readily internalized into HuD-positive cancer cells in cell culture, we hypothesized that the BW-2 immunotoxin injected directly into the tumor mass would readily recognize, enter and kill the targeted antigen-specific tumor cell. We further hypothesized that intratumoral (i.t.) immunotoxin therapy would provoke far less systemic toxicity than systemic immunotoxin therapy. In fact, one time intratumoral injection of BW-2 in subcutaneously implanted SCLC nude mice significantly inhibited local tumor growth for a 3–4 week period and prolonged duration of tumor response compared to controls, confirming the in vivo effectiveness of i.t. immunotoxin. In addition, there was no evidence of clinical toxicity in the BW-2 treated mice. Unsurprisingly, mice treated with saporin toxin alone failed to demonstrate inhibition of rapid local tumor growth, and this pattern was also observed in mice treated with anti-HuD mAb or PBS alone. All control mouse groups succumbed earlier with overwhelming local tumor growth and massive distant metastasis (Figure 4).

One of the major concerns in using anti-HuD immunotherapy in SCLC and NB is the potential development of neurologic side effects. Due to the lack of an appropriate animal model to aid in defining the precise relationship of anti-HuD humoral immunity to the pathogenesis of paraneoplastic encephalomyelitis or paraneoplastic sensory neuropathy associated with HuD-positive malignances, enthusiasm for the development of an anti-HuD antigen specific immunotherapy for patients with SCLC has remained low [11]. Similarly to DNA vaccination with mouse HuD-antigen in a mouse model of neuroblastoma, our intratumoral anti-HuD immunotoxin therapy inhibited tumor growth without inducing clinically apparent neurological symptoms in neuro-2a xenografted mice [10]. Additionally, neuropathologic examination of the brain, spinal cord and peripheral nerves in BW-2 treated mice showed no evidence of pathology as judged by presence of inflammation or neuronal death.

Elevated infiltrating cytotoxic T cells have been associated with favorable clinical outcomes in multiple tumor types [13-15]. A vaccine with DNA coding for Hu-protein in A/J mice has been reported to induce T cell infiltration to tumor sites and reduce the size of neuroblastoma (neuro-2a) tumors [10]. Our study in tumor allografted A/J mice showed that at early stages (tumor volume <600 mm^3^), substantial dendritic cells (DCs) and T cells reside within the subcutaneous tumor (data not shown). The proportion of infiltrating T cells within the tumor progressively decreases as the tumor grows, suggesting that the tumor-infiltrated immune cells may play a vital role in inhibiting local and metastasized tumor growth. The advantage of intratumoral immunotoxin therapy is that it selectively kills the targeted tumor cells and causes minimal toxicity to the immune cells within the tumor microenvironment, adjacent lymph nodes, and systemically within the body [16,17]. Furthermore, the tumor antigens released from the lysed tumor cells may be readily taken up and processed by local DCs for cross-priming [17]. The amplified activation of local anti-tumor T cells as well as T cells from other lymphoid tissues may lead to eradication of the residual and metastatic tumor cells as observed in our study.

The advent of cellular immunotherapies has further propelled the use of monoclonal antibodies in the fight against malignancies. Several anti-PD-1/anti-PD-L1 mAbs are actively in clinical trials for treatment of a variety of solid tumors including non-small cell lung cancer (NSCLC) and melanoma [18-21]. Similarly, anti-CTLA-4 agents approved for treatment of melanoma have been tested in advanced stage SCLC and NSCLC, though results for SCLC are thus far inconclusive [22,23]. The highly successful chimeric ch14.18 mAb drastically improved survival in high risk NB patients [24]. Targeting of immunologic checkpoints has become a very popular and promising approach to combating malignant disease. However, this approach relies partially on antigen presenting cell dependent immunologic responses. Our demonstrated approach may provide an excellent opportunity to catch those who fall through the cracks of the immunologic checkpoint therapies.

## Conclusions

Both SCLC and NB have very poor prognoses and 5-year survivals are less than 25% and 50% respectively despite all available aggressive multimodality therapies. Our study describing intratumoral immunotoxin therapy in mouse models of SCLC and neuroblastoma serves as a proof of concept and our findings may help to maximize immunotherapeutic efficacy for a variety of malignancies. In addition to the immunocompetent mouse model of neuroblastoma described here, the same strategy could reasonably be applied to other animal models of malignancies by switching to other tumor antigen specific immunotoxin (antibody/toxin) compounds. In the future, this new treatment may offer clinicians a novel treatment option with significant therapeutic potential for patients with solid tumors.

## Methods

### Cell lines and reagents

Human SCLC (NCI-H69, DMS-79), human erythroleukemia (K-562), mouse NB (neuro-2a) and mouse T lymphoma (BW5147) cell lines were purchased from the American Type Culture Collection (ATCC; Rockville, MD). Cells were maintained in growth medium supplemented with 10% fetal calf serum (FCS) as recommended by the vendor. Saporin (SAP) and streptavidin-SAP were purchased from Advanced Targeting Systems (Santiago, CA) and stored at −80°C until use. Biotinylated and non-biotinylated mouse anti-human HuD mAb 16A11 (IgG2b) were purchased from Molecular Probes (Eugene, OR) [8]. Immunotoxin BW-2 was prepared by premixing biotinylated-16A11 mAb with streptavidin-SAP in equal molar concentrations.

### Western blot analysis

Neuro-2a (mouse neuroblastoma) cells were lysed and total protein content was determined by measuring its absorbance. Aliquots of 50 μg total protein from each sample were subjected to SDS-polyacrylamide gel electrophoresis on a NuPAGE 4-12% Bis-Tris gel (Invitrogen, Carlsbad, CA) followed by transfer to a nitrocellulose membrane. The membrane was probed with primary antibodies anti-HuC/D (6ug/mL, Molecular Probes, Eugene, OR) followed by incubation with appropriate HRP-conjugated secondary antibodies (GE healthcare, Piscataway, NJ). The signal was visualized through the use of the ECL Plus Western Blotting Detection System (GE Healthcare) and exposure to ECL Hyperfilm (GE Healthcare). The membrane was stripped with Re-Blot Plus Strong (Chemicon, Temecula, CA).

### In vitro cell proliferation and cytotoxicity assays

Five ×10^4^ tumor cells (NCI-H69, DMS-79, neuro-2a or control K562 cells) in 100 ul of culture medium were placed in each well of 96-well plates. Log dilutions (0.005, 0.05, 0.5 and 5 μg/ml) of BW-2 were added to the cell culture wells. Equivalent molar concentrations of SAP, antibody alone as well as streptavidin alone served as controls. The cells were incubated at 37°C, 5%CO_2_ for 72 h. Cell viability was assayed using a modified tetrazolium salt 3-(4,5-dimethylthiazol-2-yl)-2,5-diphenyltetrazolium bromide assay (Cell Titer 96 Cell Proliferation Assay kit, Promega Corp., Madison, WI) according to the manufacturer’s instructions. Tunnel or trypan blue staining was used on selected cell lines to confirm the findings. Each assay was performed in triplicate.

### Animals

Male athymic nude mice at age 5–6 weeks (NCRNUM) were purchased from Taconic (Hudson, NY). Female A/J mice at 6–8 weeks were purchased from Jackson laboratory (Bar Harbor, ME). The mice were housed and maintained in specific pathogen-free conditions and the studies were conducted in accordance with the Animal Component of Research Protocol guidelines at the VA Medical Center, East Orange, NJ.

### Tumor xenograft and allografts

The mouse model of human SCLC was established by subcutaneous (s.c.) implantation of viable tumor cells (1 × 10^7^/100 μl PBS) into the flank of nude mice. Animals were monitored 5 days per week for tumor onset and growth. Tumor size was measured using calipers and final tumor volumes were calculated using a standard formula: length × width × height × 1/6 × π [9].

A mouse model of neuroblastoma was similarly established by s.c. implantation of mouse neuro-2a cells (1 × 10^7^/100 μl PBS) into the flank of immunocompetent female A/J mice at 6–8 weeks of age [10]. Tumor size was measured 5 days per week as above. When primary tumors reached approximately 150-200 mm^3^, mice (n = 5) received two intratumoral injections at weekly intervals of either BW-2 at a dose of 1 mg/kg or equivalent doses of PBS. Mice were monitored following treatments 5 times per week for tumor growth and general condition. Animals were sacrificed and perfused with 4% paraformaldahyde when tumor burden became too great.

### Treatment protocol

When subcutaneous tumor nodules reached approximately 150–200 mm^3^ in size, the SCLC tumor xenografted nude mice (n = 6) received treatment with one time intratumoral immunotoxin at doses of 1 mg/kg, or equivalent doses of pure 16A11 mAb alone, saporin toxin alone, or sham treatment with saline. Mice were monitored 5 times per week by measuring tumor size, assessing general condition, weight changes, and presence of neurological symptoms post treatment.

Minor modifications of the treatment protocol were made for the NB allografted A/J mice (n = 5). When subcutaneous NB tumor nodules reached a size of 150–200 mm^3^, mice received two intratumoral immunotoxin treatments at weekly intervals. Mice receiving equivalent doses of mAb (16A11) alone, saporin toxin alone, or sham treatment with saline served as controls. Mice were then monitored for tumor growth and general condition as described above.

### Pathologic correlation

Local tumors and organs (lung, brain, liver, adrenal gland and bone marrow) were collected for histopathologic studies at serial time points post treatment. Standard hematoxylin/eosin (H&E) and immunohistochemical reactions on the tissue sections were performed with anti-HuD antibody (16A11) to determine the extent of tumor involvement. Tissue sections of GFP-labeled neuro-2a tumors or organs that exhibited metastasis were visualized directly under fluorescent microscopy. To quantify the immune cell infiltration within the local s.c. tumor sites, immunochemical reactions on the frozen tumor tissue sections were conducted with antisera (eBioscience, San Diego, CA) specific for CD4, CD8, CD11c and Foxp3+ following manufacturer’s recommendations.

### Statistics

Statistical analysis was performed using Instat software 3.0 (Graphpad Software, Philadelphia, PA). The composite data was analyzed by the Kruskal-Wallis one-way analysis of variance and the Mann–Whitney U test (two-tailed) was used to determine the significance of intergroup clinical response differences after treatment. A value of P < 0.05 was considered significant.

## Abbreviations

SCLC: Small cell lung cancer
NB: Neuroblastoma
i.t.: Intratumoral
NSCLC: Non-small cell lung cancer
mAb: Monoclonal antibody
s.c.: Subcutaneous
